# Improved performance of stretchable piezoelectric energy harvester based on stress rearrangement

**DOI:** 10.1038/s41598-022-23005-2

**Published:** 2022-11-09

**Authors:** Young-Gyun Kim, Seongheon Hong, Bosun Hwang, Sung-Hoon Ahn, Ji-Hyeon Song

**Affiliations:** 1 Department of Mechanical Engineering, Seoul National University, Gwanak-Ro 1, Gwanak-Gu, Seoul, 08826 Republic of Korea; 2 MX Division, Samsung Electronics, Samsungro 129, Suwon-Si, Gyeonggi-Do 16677 Republic of Korea; 3 Department of Mechanical Engineering, Dankook University, Jukjeon-Ro 152, Suji-Gu, Yongin, 16890 Republic of Korea; 4 Institute of Advanced Machines and Design, Seoul National University, Gwanak-Ro 1, Gwanak-Gu, Seoul, 08826 Republic of Korea

**Keywords:** Electrical and electronic engineering, Mechanical engineering, Materials for devices

## Abstract

With the development of wearable devices and soft electronics, the demand for stretchable piezoelectric energy harvesters (SPEHs) has increased. Energy harvesting can provide energy when large batteries or power sources cannot be employed, and stretchability provides a user-friendly experience. However, the performance of SPEHs remains low, which limits their application. In this study, a wearable SPEH is developed by adopting a kirigami structure on a polyvinylidene fluoride film. The performance of the SPEH is improved by rearranging the stress distribution throughout the film. This is conducted using two approaches: topological depolarization, which eliminates the opposite charge generation by thermal treatment, and optimization of the neutral axis, which maximizes the stress applied at the surface of the piezoelectric film. The SPEH performance is experimentally measured and compared with that of existing SPEHs. Using these two approaches, the stress was rearranged in both the x–y plane and z-direction, and the output voltage increased by 21.57% compared with that of the original film with the same stretching motion. The generated energy harvester was successfully applied to smart transmittance-changing contact lenses.

## Introduction

Energy harvesters can capture and store usable energy from environments containing mechanical, thermodynamic, or electromagnetic energy sources^1^. This technology is beneficial in conditions where large batteries or power sources cannot be employed. The importance of energy harvesting is increasing in various fields, including wearable devices, renewable energy, smart sensors, future environmentally-friendly vehicles, and smart buildings^2,3^. Particularly, stretchable energy harvesters are becoming more prominent in wearable-device applications. The demand for higher performance despite the limited space within wearable devices requires the aid of energy-harvesting technology. In this situation, stretchability provides a comfortable user experience because the device can fit the curve of the human body and deform with human-body motion through mechanical compliance with strain. The energy-harvesting mechanism includes triboelectric, thermoelectric, and piezoelectric mechanisms. Among these, the piezoelectric mechanism provides high performance and is suitable for wearable devices because body motion can be regarded as mechanical strain.

Although the demand for stretchable piezoelectric energy harvesters (SPEHs) has increased for wearable devices and soft electronics, their performance is still low, which limits their application in auxiliary power sources. The main reason for the low performance of SPEHs is the decrease in the ratio of piezoelectric materials in the final product, owing to the inclusion of stretchable materials, such as polymers, to render stretchability^1,4^. However, in the form of a composite, the advantages of both materials can be utilized, i.e. the stretchability of polymer and the piezoelectric performance of piezoelectric materials. Various piezoelectric fillers, such as barium titanate and lead zirconate titanate (PZT), are integrated with polymer matrices, such as polydimethylsiloxane (PDMS), silicone rubber, and Ecoflex^5^. In addition, piezoelectric fibre^6–9^ and nanowires^10–12^ have been incorporated with stretchable substrates. Fibre and nanowires are fabricated using various methods including electrospinning^4^ and ultrasonication^13^ and have higher flexibility than that of one-dimensional nanostructures owing to their small cross-sections^1^. To further increase stretchability, three-dimensional microstructures, such as hemispheres^14,15^, pyramids^16^ and microfoams^17–19^, have been explored.

Others have attempted to use novel stretchable designs to improve stretchability in relation to that of flat structures^5,20^. This method can achieve stretchability without decreasing the piezoelectric-material-content ratio^1^. Therefore, piezoelectricity can be achieved by enduring mechanical strain. Typically, piezoelectric thin films are fabricated into stretchable structures, such as kirigami^21–28^, wavy/buckled^29–34^, serpentine^35,36^, fractal pattern^37^, helical^38^ and woven textiles structures^39–45^, to render them stretchable^5,20^. In particular, the kirigami structure has generous design freedom and an easy fabrication process unlike other methods. Stretchability is attained via distortion of the structure. However, when the structure is distorted, stress is not evenly distributed throughout it, which lowers piezoelectric efficiency. In addition, an adverse current generated from segments with opposite deflection directions decreases efficiency^46^. Therefore, SPEHs with kirigami structures are limited by low piezoelectric performance.

In this study, a wearable SPEH was developed by adopting a kirigami structure on polyvinylidene fluoride (PVDF) film. PVDF films exhibit good flexibility and biocompatibility. However, they have low piezoelectric coefficients. This study makes a unique contribution to the literature by improving the performance of SPEHs using stress redistribution within the film. This study was conducted using two approaches. The first approach uses topological depolarization, which eliminates the generation of opposite charges and increases the overall piezoelectric performance. The second approach uses a backing layer to optimize the neutral axis and maximize the stress applied to the surface of the piezoelectric film. The SPEH performance was measured and compared to that of other devices. Finite element analysis (FEM) was conducted to determine the kirigami pattern design that produced the highest average stress within the constraints. Finally, the developed SPEH was successfully applied in smart transmittance-changing contact lenses.

The remainder of this paper is organized as follows. The concept of stress rearrangement with topological depolarization and neutral-axis optimization is examined. Then, the output voltage is compared with and without the two approaches, and their effects are investigated. The results are compared with those of other studies on SPEHs with a novel stretchable design, and the application of the SPEH is also introduced. Subsequently, conclusions, limitations, and future work regarding this study are summarized. Finally, the methods and materials used to fabricate the SPEH are presented.

## Results

### Concept of stress-rearranged stretchable piezoelectric energy harvester

**Figure 1:**
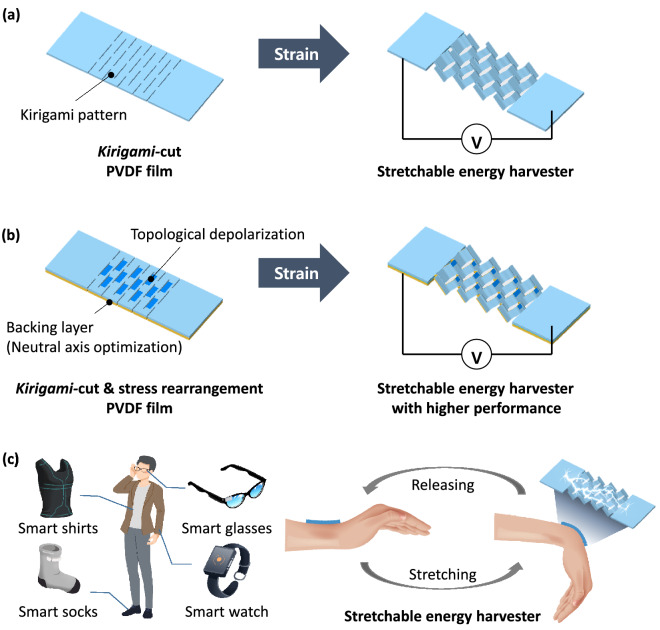
Schematic of the stress-rearranged stretchable piezoelectric energy harvester. (**a**) Stretchable energy harvester using kirigami pattern, (**b**) high-performance stretchable energy harvester using stress rearrangement and (**c**) high-performance energy harvester for wearable-device applications.

Figure 1 shows the concept of a stress-rearranged SPEH. A kirigami pattern is adopted to attain stretchability in the rigid PVDF film, as shown in Fig. 1a. When stretched, stress is applied to the film and generates an electrical charge, which can be collected and utilized. Figure 1b shows the concept of a high-performance SPEH that uses stress rearrangement. Stress distributed via stretching can be controlled using two approaches: topological depolarization and neutral-axis optimization using a backing layer. By rearranging the stress distribution, the direction of the generated charge becomes consistent, and the stress applied to the surface of the piezoelectric film increases, which results in higher piezoelectric performance. A detailed theoretical description of how these approaches increase piezoelectric performance is presented in the discussion. Due to its improved performance, the developed SPEH can be successfully adopted in wearable applications, as shown in Fig. 1c. It can be applied to the wrist, and the bending motion of the wrist can generate energy, which can be used as a power supply for wearable devices. The improved performance enables the successful implementation of a SPEH on a wearable device without a large number of stretching motions or time delays, thereby providing a user-friendly experience.

### Effect of topological depolarization

**Figure 2:**
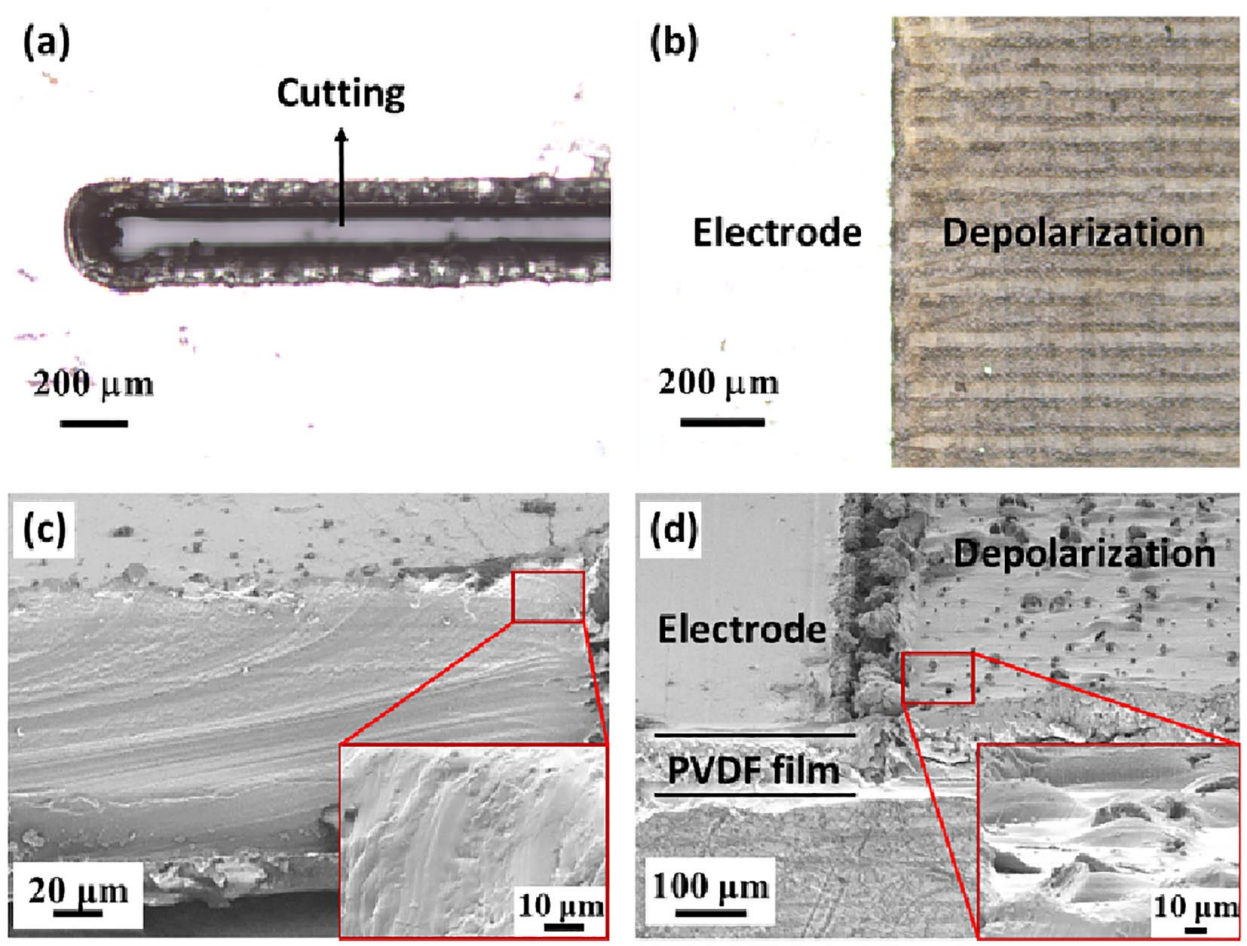
(**a**–**b**) Optical images of cutting and depolarization area. SEM images of (**c**) laser-cut section and (**d**) electrode-coated and depolarized surface. The SEM image was tilted by 25°.

Figure 2a–d shows the optical and scanning electron microscopy (SEM) images of the cutting and depolarized areas of PVDF film. As shown in Fig. 2a,b clear interlayers were observed for cutting and depolarization. The size of the depolarization region was determined using the FEM analysis result. The tensile and compressive stresses are generated according to the concave and convex bending motions of the segments, respectively. Therefore, the output voltage is maximized by performing thermal depolarization of the area with different bending directions using a laser. The resolution of the laser equipment and the size of heat affected zone were considered. The minimum cutting resolution was 80 μm, which was the laser beam diameter. The heat-affected zone (HAZ) of the laser was 100 um. Therefore, we maintained a minimum width of 0.5 mm between the cutting lines for durability. The geometry of the cutting edge in the laser-cutting process was round, which prevents stress concentration; therefore, the samples can endure a higher strain than those of the sharp edge produced by a cutter. The surface of the depolarized area was dark because the electrode layer was removed during depolarization. The surface and cross-section of the cut and depolarized areas were observed using SEM with a 25° tilting, as shown in Fig. 2c,d. The surface of the PVDF material affected by the laser thermal energy used for cutting is observed in the cross-sectional image shown in Fig. 2c. Figure 2d shows the electrode-coated and depolarized areas. The results indicated a difference in the surface roughness of the films. As shown in the magnified image, the horizontal lines appear periodically differentiated by the remaining structures, owing to the laser fabrication path.

**Figure 3:**
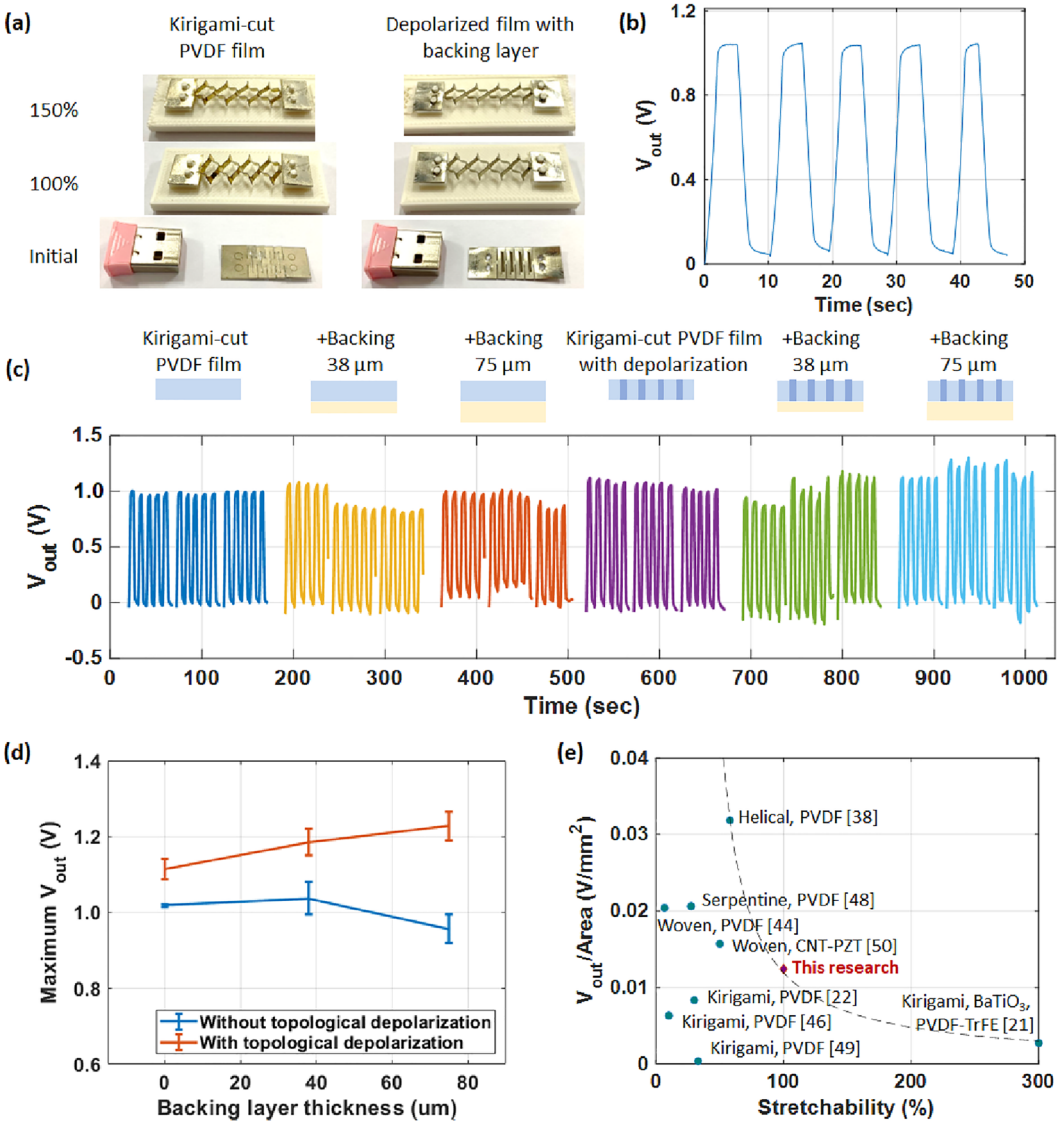
Electromechanical analysis of the (**a**) stretching results of the kirigami-cut PVDF film and the topological depolarized film with a backing layer attached using 100% and its maximum stretchability, (**b**) output voltage–time plot, (**c**) output voltage with time, (**d**) maximum output voltage of samples with different conditions, and (**e**) comparison of stretchability and voltage per unit area with results of other studies.

Figure 3a shows an isometric view of the stretched results of the kirigami-cut PVDF film and topologically depolarized film with a backing layer attached. The film with depolarization and a backing layer exhibited the same maximum stretchability as that of the kirigami-cut PVDF film. The observed deformation was the same as that observed in the FEM analysis which can be found in the Supplementary Information. Stretchability was achieved via the convex and concave bending motions of the segments. The maximum stretchability was 150%; however, 100% was applied for electromechanical analysis.

Figure 3b–c shows the output voltage–time plot achieved with stretching and releasing motions. The voltage graph shows stable maintenance of the voltage peak due to the designed circuit^47^. The kirigami-cut PVDF film, the film with a backing layer of 38 and 75 µm, the kirigami-cut PVDF film with topological depolarization, and that with a backing layer of 38 and 75 µm were compared. Three samples were tested for each condition, and each test was repeated five times. The average output voltage and standard deviation over time are shown in the Supplementary Information. The maximum average output voltages under different conditions are shown in Fig. 3d.

A comparison of the kirigami-cut PVDF films with and without topological depolarization shows that the output voltage increased from 1.02 to 1.14 V, in other words, an improvement of 11.76%. Samples with 38 and 75 µm backing layers also exhibited an improvement of 13.33% and 29.17% with depolarization, respectively. The PVDF film was placed in an oven at 120 °C for 2 h to evaluate the thermal depolarization effect. The film was cut using the same kirigami pattern, and the output voltage was measured. The results exhibited a piezoelectric performance that was decreased by 38.56%, which is similar to the results of the laser treatment. The corresponding data are available in the Supplementary Information. Therefore, we can expect that sufficient thermal energy was provided for thermal depolarization with the laser process.

### Effect of neutral-axis optimization

With topological depolarization, the films with backing layers with thicknesses of 38 and 75 µm exhibited output voltages of 1.19 and 1.24 V that increased by 4.39% and 8.77%, respectively. However, without topological depolarization, the film with a backing layer with a thickness of 38 µm exhibited an output voltage that increased by 2.94%, whereas that with 75 µm exhibited a decreasing trend. This is owing to the difference in Young’s modulus values of the depolarized film. When the film was depolarized, Young’s modulus of the film decreased owing to the thermal effect and the electrode removal. The backing layer improves the piezoelectric performance; however, a thicker film results in a higher Young’s modulus, and it eventually decreases the piezoelectric effect, owing to the difficulty in obtaining mechanical compliance with strain. Samples with depolarization had a lower Young’s modulus than the original film; therefore, a thicker backing layer produced a higher output voltage, and a backing layer of 75 µm still had a positive effect. However, films without depolarization had a higher Young’s modulus than that of the depolarized film; therefore, Young’s modulus of the composite with a backing layer was higher. A 38-µm backing layer increased the piezoelectric efficiency, whereas a thicker backing layer of 75 µm had a negative effect. As a result, the samples with topological depolarization and a backing layer of 75 µm exhibited the highest output voltage, which was 21.57% higher than that of the kirigami-cut PVDF film.

### Comparison

Figure 3e shows a comparison of the film stretchability and output voltage per unit area^5^. Other reported studies of SPEHs based on stretchable structures were compared with the developed SPEH. Zhou et al. reported the highest stretchability of 300% using BaTiO_3_ and Polyvinylidene fluoride-trifluoroethylene-chlorofluoroethylene (PVDF-TrFE) composites in kirigami structures^21^. However, the developed device had a relatively low output voltage of 0.0027 V/mm^2^. Kim and Yun reported the highest output voltage per unit area of 0.0318 V/mm^2^ using a PVDF strap wired in a helical structure on an elastic core^38^; however, the maximum stretchability was relatively low (58%). The developed SPEH has a stretchability of 100% (maximum stretchability of 150%) and a voltage per unit area of 0.0124 V/mm^2^; thus, it exhibits the advantages of both stretchability and voltage generation. A detailed comparison of the materials, structures, stretchability, and output voltages obtained in other studies is listed in Table 1.

**Table 1:** Comparison of SPEHs based on stretchable structures^5^.

Structure	Materials	Stretchability (%)	Output voltage (V)	Output voltage/Area (V/mm^2^)	Ref
Buckled	PVDF	30	0.04	–	^30^
Serpentine	PVDF	27.5	9.9	0.0206	^48^
Kirigami	PVDF	10	1.63	0.0063	^46^
Kirigami	PVDF	33	0.333	0.0004	^49^
Kirigami	PVDF	30	14	0.0083	^22^
Kirigami	BaTiO_3_, PVDF-TrFE	300	6	0.0027	^21^
Helical	PVDF	58	75	0.0318	^38^
Woven	PVDF	6.7	51	0.0204	^44^
Woven	CNT-PZT	50	5.65	0.0157	^50^
Woven	PVDF	58	0.38	–	^51^
Kirigami	PVDF	100	1.2	0.0120	This study

### FEM analysis of kirigami pattern design for applications

**Figure 4:**
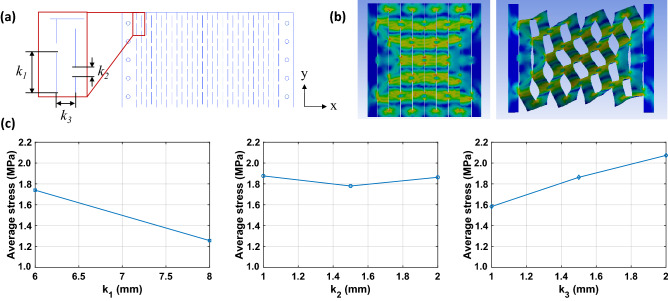
FEM analysis for application design. (**a**) design parameters for kirigami pattern, (**b**) captured image of the stress applied to the film, and (**c**) averaged stress with respect to design parameters identified using the Taguchi method.

Multiple patterns with 4-5-4 cuts were employed for applications. These patterns produced higher stress than those of the 1-2-1 cuts. A FEM analysis (ANSYS, Ansys Inc., USA) was conducted to determine the kirigami design parameters that produce the highest stress with stretching. A size and maximum stretchability of 70 $\times$ 46.5 mm^2^ and 30%, respectively, were selected as the constraints because these are the required values for wrist applications. The design parameters were adjusted based on these constraints. Figure 4 shows the design parameters and FEM analysis for the kirigami patterns. As shown in Fig. 4a, the width of the cutting and the gap between the cuts in the x- and y-directions are denoted as k_1_, k_2_ and k_3_, respectively. The FEM analysis conditions using the Taguchi method^52^ are listed in Table 2. Figure 4b,c show the results of the FEM analysis. As parameter k_1_ increased, the average stress decreased. Changes in parameter k_2_ induced little difference in the stress. When k_3_ increased, the stress also increased. Therefore, condition 2, for which k_1_, k_2_, and k_3_, are 6, 1.5 and 2.5 mm, respectively, was identified to produce the highest stress according to the Taguchi method. These parameters were selected for the application design.

**Table 2:** FEM analysis conditions using Taguchi method.

Condition no	Width of the cut, k_1_	Gap in x-axis, k_2_	Gap in y-axis, k_3_
1	6	1	2
2	6	1.5	2.5
3	6	2	1.5
4	8	1	2.5
5	8	1.5	1.5
6	8	2	2

### Applications: smart transmittance-changing contact lens

**Figure 5:**
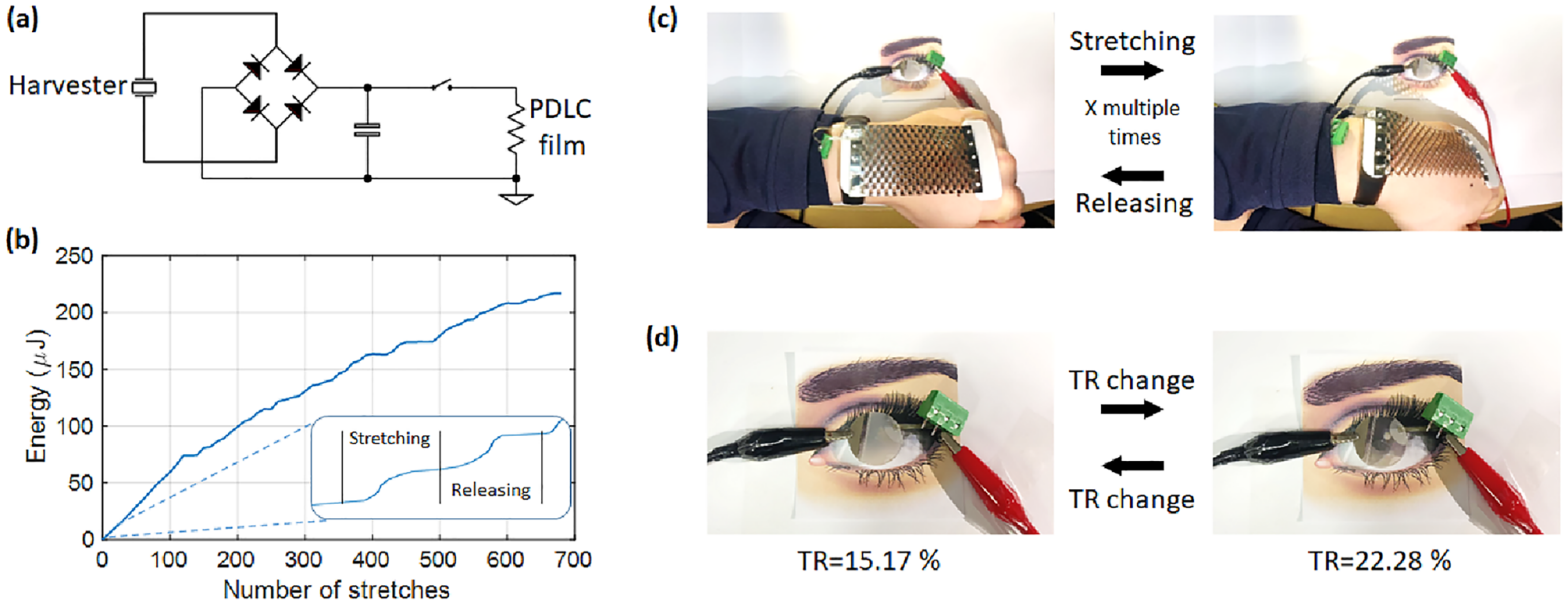
Transmittance-changing contact lens (**a**) Energy-harvesting circuit including capacitor and diode, (**b**) harvested energy with respect to the number of stretches, (**c**) stretching and releasing motion of SPEH applied on the wrist, and (**d**) TR changing of PDLC films with harvested energy.

Figure 5 shows a transmittance-changing contact lens with the voltage generated by a developed SPEH. Figure 5a shows the circuit that connects the lens and SPEH. A capacitance of 4.7 μF is connected to the diode. Figure 5b shows the energy generated according to the number of stretching and releasing motions. One bending motion can generate a maximum of 7.83 µJ and an average of 3.24 µJ. Range of the wrist motion was 22.2° of extension to 59.8° of flexion and the frequency was 1.73 Hz. The generated energy exhibited a saturation trend as the number of stretches increased, owing to the limitations of the circuit. The diode always had a current leakage, even though a commercial diode with a low current leakage of 10 pA was used. The diode can have a reverse voltage of up to 15 V, which means that the maximum voltage stored in the capacitor cannot exceed 15 V. With an increase in the stored voltage within the capacitor, the possible range of harvesting decreases. The voltage can be collected when it is higher than the sum of voltage within the capacitor and the forward voltage of the diode. Therefore, the efficiency of the SPEH decreased as the energy stored in the capacitor increased. The aging of the SPEH may provide another reason for the decrease in efficiency. Figure 5c shows the SPEH adopted for the wrist and its bending motion. The energy generated by stretching and releasing is stored in the capacitor with the capacitance of 1 μF, which changes the transmittance (TR) of the polymer-dispersed liquid crystal (PDLC) film. Figure 5d shows the PDLC film before and after the transmittance change. The transmittance was measured using a spectrometer (Frame S, Ocean optics, USA). In the visible wavelength range, the transmittance increased from 15.17 to 22.28%. At 550 nm, the transmittance changes from 13.22 to 19.98%.

The energy can be stored up to 15 V with 100 repeated stretching and releasing cycles with the 1 μF capacitor. The stored energy is dissipated due to the resistance of the lens (200 MΩ). Therefore, the transmittance of the PDLC film could be changed for 14 s after 100 repeated stretching and releasing cycles according to the equation $t=-RC\mathrm{ln}\frac{V}{{V}_{0}}$. As presented in the aforementioned section, the developed SPEH has a high energy density; therefore, only a small amount of stretching is required to change the transmittance of the contact lens. Other developed SPEHs must stretch and release multiple times (hundreds or thousands of times) to charge, which is not practical for real-life applications. A video of the transmittance change of the contact lens is included in the Supplementary Information (Supplementary Video). This application can be utilized in self-powered smart sunglasses.

## Discussion

**Figure 6:**
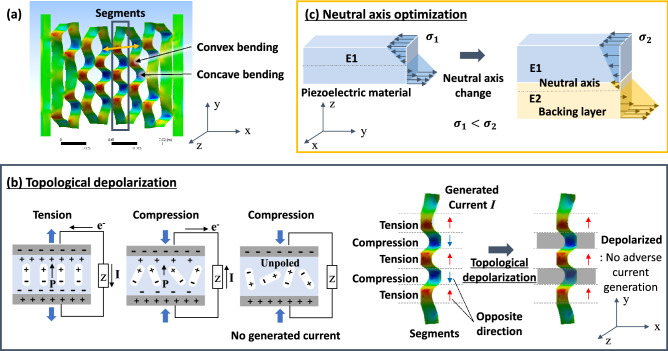
Concept of stress rearrangement used to increase piezoelectric performance. (**a**) FEM results showing the kirigami pattern deformation, (**b**) principle of topological depolarization and (**c**) neutral-axis optimization.

Figure 6 shows the concept of stress rearrangement used to increase the piezoelectric performance of a SPEH. Figure 6a shows the FEM analysis of the kirigami pattern when stretched. The structural deformations of concave and convex bending were mixed to attain stretchability, and they are indicated in blue and red, respectively. Figure 6b shows the concept of topological depolarization, and Fig. 6c depicts the concept of using neutral-axis optimization to rearrange the stress over the film and improve piezoelectric performance. Topological depolarization rearranges the stress in the x- and y-directions of the film and the neutral-axis optimization rearranges the stress in the z-direction.

### Topological depolarization

In the piezoelectric film, if the piezoelectric poling is oriented in the same direction, tension and compression produce currents in different directions, as shown in Fig. 6b. However, in an unpoled material, the dielectric is not oriented in a single direction; therefore, compressive and tensile stress cannot generate an electric charge. Thermal depolarization can occur when the temperature of the film exceeds 74 °C, where dipoles rotate away from their originally poled direction^53^. The actual temperature of the film measured by a thermal imaging camera (Hanmac, HM-300 with a spectral range of 8–14 μm) was 124.67 °C.

Kirigami patterns are considered to be the combination of the segments marked in the blue square shown in Fig. 6a. Convex and concave bending result in the tension and compression of the piezoelectric film on the surface, as shown in Fig. 6b. As explained, the generated current is in different directions. If we remove the current generation in the opposite direction via depolarization and only leave the current in the same direction, the total current will increase. In this manner, the stress distribution on the surface of the film in the x- and y-directions is rearranged, thereby resulting in higher piezoelectric performance. In this study, laser irradiation was used for the topological depolarization of the film based on FEM analysis.

### Neutral-axis optimization

As shown in Fig. 6c, the neutral axis was optimized to change the stress in the z-direction using a backing layer. Assume that the width of the cross-section is *b*, the thickness and Young’s modulus of the PVDF film are *h*_*1*_ and *E*_*1*_, respectively, and those of the backing layer are *h*_*2*_ and *E*_*2*_. For simplicity, the adhesion layer was not considered because it is relatively thin compared to the PVDF film and backing layer. Based on Euler–Bernoulli beam theory and transformed-section method, the vertical centroid and second moment of inertia of the structure can be calculated as follows^54^:

1 $$\overline{y }=\frac{{2E}_{1}{{h}_{1}h}_{2}+{E}_{1}{{h}_{1}}^{2}+{E}_{2}{{h}_{2}}^{2}}{{2E}_{1}{h}_{1}+{2E}_{2}{h}_{2}},$$

2 $${I}_{tot}=\frac{1}{12}b{{h}_{1}}^{3}+b{h}_{1}{\left(\frac{{E}_{2}{{h}_{2}}^{2}+{E}_{2}{{h}_{1}h}_{2}}{{2E}_{1}{{h}_{1}+{2E}_{2}h}_{2}}\right)}^{2}+\frac{1}{12}\frac{{E}_{2}}{{E}_{1}}b{{h}_{2}}^{3}+\frac{{E}_{2}}{{E}_{1}}b{h}_{2}{\left(\frac{{E}_{1}{{h}_{1}}^{2}+{E}_{1}{{h}_{1}h}_{2}}{{2E}_{1}{{h}_{1}+{2E}_{2}h}_{2}}\right)}^{2}.$$

For simplification, the deformation of each segment of the kirigami structure due to stretching can be regarded as a symmetric bending with concentrated load P and both ends fixed.

Without the backing layer, the maximum deflection ${\delta }_{max1}$ and stress ${\sigma }_{1}$ can be calculated as follows^54^:

3 $${\delta }_{max1}=\frac{{P}_{1}{L}^{3}}{192{E}_{1}{I}_{1}},$$

4 $${\sigma }_{1}=\frac{\left(\frac{{P}_{1}L}{8}\right)\left(\frac{{h}_{1}}{2}\right)}{{I}_{1}}=\frac{{P}_{1}L{h}_{1}}{16{I}_{1}},$$

where *L* is the length, and *I*_*1*_ is the moment of inertia of the sample. Using the backing layer, the maximum deflection ${\delta }_{max2}$ and stress ${\sigma }_{2}$ are calculated as follows:

5 $${\delta }_{max2}=\frac{{P}_{2}{L}^{3}}{192{E}_{1}{I}_{tot}},$$

6 $${\sigma }_{2}=\frac{\left(\frac{{P}_{2}L}{8}\right)\left({h}_{1}+{h}_{2}-\overline{y }\right) }{{I}_{tot}},$$

In the stretching experiments, the maximum deflection was the same for both cases. Therefore,

7 $${P}_{2}={P}_{1}\frac{{I}_{tot}}{{I}_{1}}\frac{{\delta }_{max2}}{{\delta }_{max1}},$$

8 $${\sigma }_{2}=\frac{{P}_{1}L}{16{I}_{1}}\left(\frac{{E}_{1}{{h}_{1}}^{2}+2{E}_{2}{{h}_{1}h}_{2}+{E}_{2}{{h}_{2}}^{2}}{{E}_{1}{{h}_{1}+{E}_{2}h}_{2}}\right).$$

In piezoelectric materials, the strain–charge form can be expressed as^55^

9 $${D}_{3}\left(x,y\right)={\varepsilon }_{33}^{T}{E}_{3}+{d}_{31}\sigma \left(x,y\right),$$

where ${D}_{3}$ and ${E}_{3}$ are the displacement and electric field in the z-axis direction, respectively, and $\sigma$ is the stress in the x-direction. ${\varepsilon }_{33}$ is permittivity, and ${d}_{31}$ is piezoelectric constant.

For an open circuit, the dielectric displacement ${D}_{3}$ is zero; therefore^55^,

10 $${\varepsilon }_{33}^{T}{E}_{3}=-{d}_{31}\sigma \left(x,y\right).$$

From the definition of uniform electric field strength,

11 $${E}_{3}=\frac{{V}_{oc}}{{t}_{p}},$$

where ${t}_{p}$ is the thickness of the piezoelectric material, and ${V}_{oc}$ is the open-circuit voltage. Therefore, ${V}_{oc}$ can be expressed as:

12 $${V}_{oc}=-\frac{{t}_{p}{d}_{31}}{{\varepsilon }_{33}^{T}}\sigma \left(x,y\right).$$

Here ${V}_{oc}$ is proportional to the stress $\sigma (x,y)$ at the surface of the piezoelectric material.

If we compare the stress with and without a backing layer, which are described using Eqs. (4 and 8), we can analyse the effect of the neutral axis on the output voltage. By substituting the values ${E}_{1}\cong 0.8 GPa$, ${E}_{2}\cong 3 GPa,$ and ${h}_{1}=80 \mu m$ and calculating ${\sigma }_{2}$ with respect to *h*_*2*_, we can observe that the calculated values are always larger than ${\sigma }_{1}$. When the backing layer is attached, the stress on the surface increases, and the output voltage increases according to Eq. (12). The value of ${\sigma }_{2}$ increases as the value of *h*_*2*_ increases. However, we cannot increase the thickness of the backing layer infinitely because a thicker backing layer increases the stiffness of the whole film; therefore, deforming the structure becomes difficult.

## Conclusions

In this study, a high-performance SPEH was developed. A kirigami pattern was adopted on flat PVDF film to render it stretchable. Higher performance can be achieved by rearranging the stress throughout the film using two approaches. The first approach uses stress redistribution over the x–y plane using thermal topological depolarization with a laser process. When the kirigami pattern stretches, concave and convex bending are mixed, which results in the generation of currents in different directions. Topological depolarization enables the current to remain in the same direction, thereby increasing the overall output voltage. The second approach rearranges the stress in the z-direction by optimizing the neutral axis. The change in the neutral axis, using the attachment of a backing layer, increases the stress applied to the surface of the piezoelectric film. Therefore, the output voltage increased. The theoretical background of this study was analysed, and the effects of topological depolarization and neutral-axis optimization were evaluated. When these two approaches were combined, the output voltage exhibited a maximum increase of 21.57%. The developed energy harvester also provided the advantages of both high output voltage and stretchability compared to the devices developed in related studies. Due to the improved performance, the developed piezoelectric energy harvester can be successfully applied to smart transmittance-changing contact lenses, which can be used as wearable sunglasses. With 100 stretch-and-release cycles, the transmittance of the lens changed from 15.17 to 22.28%.

This study can be further developed in the future. Different kirigami patterns can be introduced to achieve higher applied stress and stretchability. The piezoelectric mode used in the kirigami SPEH was the d_31_ mode. However, the PVDF film exhibited the highest piezoelectric coefficient in the d_33_ mode. The performance of the energy harvester can be improved via electrode design or a novel pattern design to apply stress to the film, the same with the poling direction. In terms of application, the contact lens can be wirelessly connected to the energy harvester for real-life applications. In this study, the circuit used to store energy hinders the continuous storage of the harvested energy. The circuit design can be improved to achieve higher efficiency.

## Materials and methods

### Fabrication of stretchable piezoelectric energy harvester

A PVDF film (Fils Co., Ltd., South Korea), uniaxially stretched and poled with a field of 1 MV/cm was used in this study. The thickness of the film was 80 µm. Poly (3,4-ethylenedioxythiophene) polystyrene sulfonate (PEDOT:PSS), carbon nanotube (CNT), and silver were used as electrode materials (thickness: 200 nm). To improve the durability of the electrode, a PEDOT:PSS layer was coated on the PVDF to flatten the surface, and a silver thin film was coated on top of it.

A laser process under different conditions was used for fabrication, which included the cutting of the kirigami pattern and thermal depolarization. At particular laser-power, damage to the film surface begins to occur. When the laser-power is increased, the films are subsequently cut. Between these two energy densities, there is a laser-power range for which the film is not cut; however, thermal damage occurs in the film, which results in the thermal depolarization of the PVDF film. During this process, the electrode is also removed. For the design of the kirigami cutting and depolarization: 1-2-1 cutting was used for parameter studies, and depolarization was conducted. The sizes of the whole kirigami pattern and depolarized area are $10\times 10 {\mathrm{mm}}^{2}$ and $6 \times 0.625 {\mathrm{mm}}^{2},$ respectively. This area was selected from the FEM analysis, the results of which are presented in the Supplementary Information. Thermal depolarization was conducted using a fibre nanosecond laser with a wavelength of 1042 nm (Inya, Inlaser, South Korea). The laser power, frequency, scan speed, and scan number were controlled to obtain the appropriate laser energy densities for depolarization. The applied power was 18 W, and the frequency was 100 Hz. The scan speed and number were 1000 mm/s and 1, respectively. Cutting was conducted using a CO_2_ continuous-wave (CW) laser (FCS, Inlaser, South Korea) with a wavelength of 10,600 nm at 250 W, and the scan speed and number of scans were 300 mm/s and 1, respectively.

Samples with and without depolarization were attached to backing layers of different thicknesses for analysing the effect of neutral-axis optimization. A polyethylene terephthalate (PET) film (38 and 75 µm thickness) with acrylic adhesive on one side (10 µm thickness) was used as the backing layer and attached to the electrode-coated PVDF film. The Young’s modulus of the PET film was 3 GPa. The position of the neutral axis is greatly influenced by the Young's modulus. PET has a relatively high modulus among low-cost polymer films (including polyethylene, thermoplastic polyurethane, and polypropylene).

### Measurement system

The stretching/releasing test and measurement system was prepared as shown in Figure S4. A data acquisition (DAQ, NI-6009, National Instruments, USA) was used to measure the output voltage of the film with the designed circuit^47^. It was automatically operated using Labview (National Instrument, NI). The sample was stretched to 100% with a strain rate of 5 mm/s. The circuit consisted of an op-amp voltage follower and capacitor for accurately measuring the output voltage. The capacitance of the circuit was 4350 pF. The parallel resistance included in the DAQ measurement circuit was removed to prevent charge loss, which enabled a stably generated charge^47^.

### Fabrication of smart transmittance-changing contact lens

The PDLC film (Glart Inc., South Korea) with a thickness of 0.5 mm was used for a smart transmittance-changing contact lens. The PDLC film has a sandwich structure with an indium tin oxide (ITO) electrode ($150\Omega /{cm}^{2}$) and PET film for protective purposes. The driving voltage was 40–100 VAC, and the power consumption was 5 W/m^2^. The PDLC film was laser-cut into a circular shape with a diameter of 20 mm. The film was then wired using an anisotropic conductive film. Thermoforming was conducted at 90 °C to transform the film into a contact lens shape with a curve radius of 30 mm.

## Supplementary Information

Supplementary Video 1.

Supplementary Figures.

## Data Availability

The datasets used and/or analysed during the current study available from the corresponding author on reasonable request.
