# Author Correction: Improved performance of stretchable piezoelectric energy harvester based on stress rearrangement

**DOI:** 10.1038/s41598-022-25906-8

**Published:** 2022-12-09

**Authors:** Young‑Gyun Kim, Seongheon Hong, Bosun Hwang, Sung‑Hoon Ahn, Ji‑Hyeon Song

**Affiliations:** 1 Department of Mechanical Engineering, Seoul National University, Gwanak‑Ro 1, Gwanak‑Gu, Seoul, 08826 Republic of Korea; 2 MX Division, Samsung Electronics, Samsungro 129, Suwon‑Si, Gyeonggi‑Do 16677 Republic of Korea; 3 Department of Mechanical Engineering, Dankook University, Jukjeon‑Ro 152, Suji‑Gu, Yongin, 16890 Republic of Korea; 4 Institute of Advanced Machines and Design, Seoul National University, Gwanak‑Ro 1, Gwanak‑Gu, Seoul, 08826 Republic of Korea

Correction to: *Scientific Reports* https://doi.org/10.1038/s41598-022-23005-2, published online 09 November 2022

In the original version of this Article, Sung-Hoon Ahn was incorrectly affiliated with ‘MX Division, Samsung Electronics, Samsungro 129, Suwon-Si, Gyeonggi-Do, 16677, Republic of Korea’.

The correct affiliations are listed below.

Department of Mechanical Engineering, Seoul National University, Gwanak-Ro 1, Gwanak-Gu, Seoul, 08826, Republic of Korea

Institute of Advanced Machines and Design, Seoul National University, Gwanak-Ro 1, Gwanak-Gu, Seoul, 08826, Republic of Korea

The original Article has been corrected.

