# Supplementary material for: Improved performance of stretchable piezoelectric energy harvester based on stress rearrangement
Source: Sci Rep. 2022 Nov 9;12:19149. doi: 10.1038/s41598-022-23005-2 (PMC9646885; doi:10.1038/s41598-022-23005-2)
Supplement: Supplementary file 2 — Supplementary Figures. [file 41598_2022_23005_MOESM2_ESM.doc]

**Improved Performance of Stretchable Piezoelectric Energy Harvester Based on Stress Rearrangement**

**Young-Gyun Kim**^1,2,+^**, Seongheon Hong**^1,+^**, Bosun Hwang^3^, Sung-Hoon Ahn**^2,4,*^**, and Ji-Hyeon Song**^3,*^

^1^Department of Mechanical Engineering, Seoul National University, Gwanak-ro 1, Gwanak-gu, Seoul, 08826, Republic of Korea

^2^MX Division, Samsung Electronics, Samsungro 129, Suwon-si, Gyeonggi-do, 16677, Republic of Korea

^3^Department of Mechanical Engineering, Dankook University, Jukjeon-ro 152, Suji-gu, Yongin, 16890, Republic of Korea

^4^Institute of Advanced Machines and Design, Seoul National University, Gwanak-ro 1, Gwanak-gu, Seoul, 08826, Republic of Korea

[*ahnsh@snu.ac.kr](mailto:*ahnsh@snu.ac.kr), [jhsong@dankook.ac.kr](mailto:jhsong@dankook.ac.kr)

^+^these authors contributed equally to this work

**SUPPLEMENTARY INFORMATION**

1. FEM analysis

Figure S1 shows the FEM analysis of the kirigami-cut PVDF film with convex and concave bending areas. The topological depolarization area was selected based on this analysis.

Figure S1. (a) Captured image of FEM results of the kirigami-cut PVDF film, and (b) averaged stress with strain

1. Electromechanical analysis

Figure S2 shows the average output voltage obtained via stretching of the samples with and without depolarization and with backing layers of 38 and 75 µm. The stage was rested for 2 s before the release.

Figure S2. Average output voltage with time of (a) kirigami-cut PVDF film, (b) kirigami-cut PVDF film with 38 µm, and (c) 75 µm backing layer, (d) kirigami-cut PVDF film with depolarization, (e) kirigami-cut PVDF film with depolarization with 38 µm, and (f) 75 µm backing layer

1. Effect of thermal treatment for depolarization

Figure S3 shows a comparison of the output voltages with and without the thermal treatment using laser treatment and oven curing process. The figure shows a decrease in the piezoelectric performance compared to the original film.

Figure S3. Comparison of the output voltage with and without thermal treatment using laser treatment and oven curing process

4. Application: smart transmittance-changing contact lens

A video of the transmittance change of the contact lens is attached (Supplementary Video).

5. Experiment Setup

Figure S4 shows the experimental setup of the application (smart transmittance-changing contact lens) and the experimental procedure.


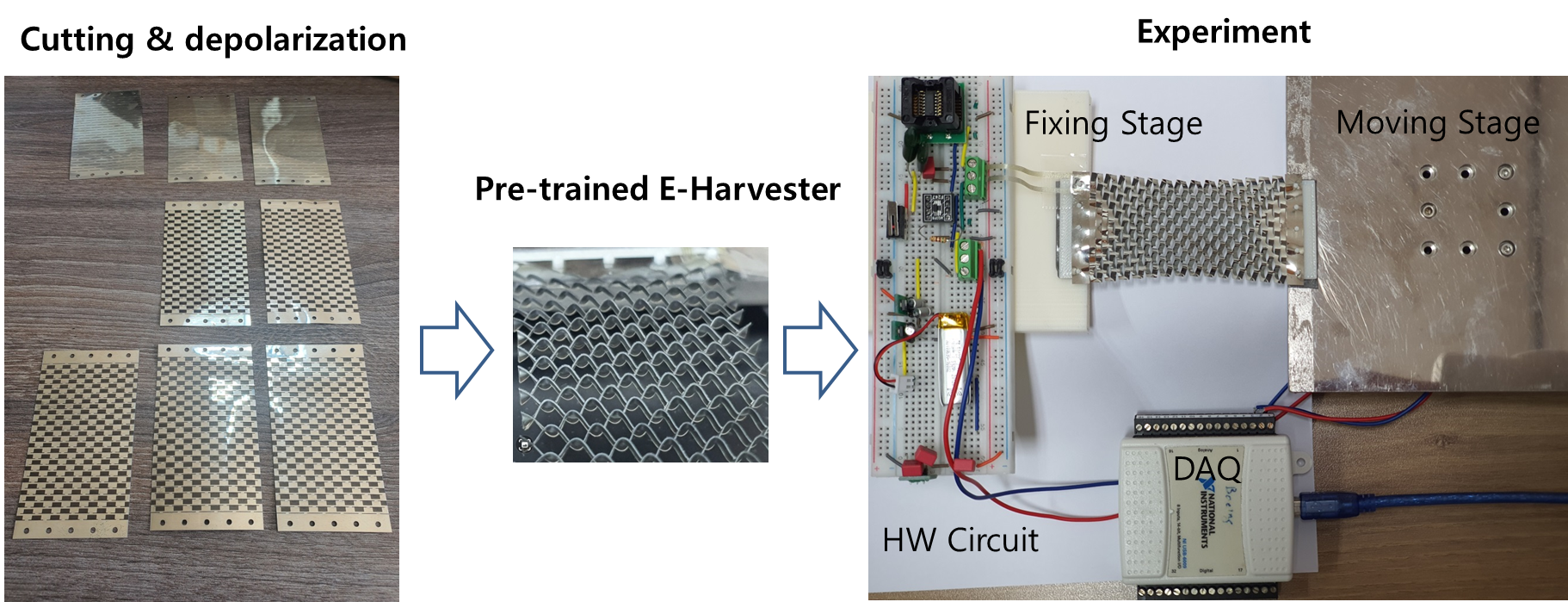


Figure S4. Experiment setup of application: smart transmittance-changing contact lens
